# Application of Magnetic Resonance Techniques to the In Situ Characterization of Li-Ion Batteries: A Review

**DOI:** 10.3390/ma13071694

**Published:** 2020-04-04

**Authors:** Sergey Krachkovskiy, Michel L. Trudeau, Karim Zaghib

**Affiliations:** Center of Excellence in Transportation, Electrification and Energy Storage, Hydo-Québec, 1806 Bd. Lionel-Boulet, Varennes, QC J3X 1S1, Canada; Krachkovskiy.Sergey@hydro.qc.ca (S.K.); trudeau.michel@hydro.qc.ca (M.L.T.)

**Keywords:** NMR, MRI, Li-ion batteries, electrolyte, anode, cathode

## Abstract

In situ magnetic resonance (MR) techniques, such as nuclear MR and MR imaging, have recently gained significant attention in the battery community because of their ability to provide real-time quantitative information regarding material chemistry, ion distribution, mass transport, and microstructure formation inside an operating electrochemical cell. MR techniques are non-invasive and non-destructive, and they can be applied to both liquid and solid (crystalline, disordered, or amorphous) samples. Additionally, MR equipment is available at most universities and research and development centers, making MR techniques easily accessible for scientists worldwide. In this review, we will discuss recent research results in the field of in situ MR for the characterization of Li-ion batteries with a particular focus on experimental setups, such as pulse sequence programming and cell design, for overcoming the complications associated with the heterogeneous nature of energy storage devices. A comprehensive approach combining proper hardware and software will allow researchers to collect reliable high-quality data meeting industrial standards.

## 1. Introduction

Global interest in finding and developing alternative energy sources is at an all-time high. The widespread adoption of electric vehicle (EV) technology, which can reduce the use of fossil fuels in urban centers, has attracted significant attention. EVs are considered to be environmentally friendly for multiple reasons, including the greater efficiency of electric motors compared to internal combustion engines and the utilization of renewable and emission-free energy sources. The governments of several countries have already announced upcoming bans of gas- and diesel-powered cars [1]. Bottlenecks to the widespread availability of EVs include the cost, capacity, safety, and dynamic performance of portable electric energy production systems, particularly batteries. Industrial targets for batteries that are appropriate for automotive applications include superior charging rates (80% change in state of charge within 15 min), extended lifetimes (>1000 cycles at 80% depth of discharge), extended operating temperature ranges (−40 to +65 °C), reliability, safety, and cost reduction [2]. Additionally, there are also long-term targets related to energy and power density.

The optimization of existing battery materials (cathodes, anodes, electrolytes) and development of alternative chemistries with superior properties rely heavily on characterization techniques that are capable of accurately detecting and quantifying the mechanisms of cell failure, including the identities of short-lived chemical species, and changes as functions of material properties, cycling rates, cell temperatures, and lifetimes. Nuclear magnetic resonance (NMR) spectroscopy is a method that has attracted significant attention over the past decade based on its ability to study a range of phenomena in operating energy storage devices in situ [3,4,5,6,7]. The main benefits of this technique over alternative approaches stem from its non-destructive nature and applicability to both crystalline and amorphous materials. The spin angular momentum of some nuclei (e.g., ^1^H, ^6,7^Li, ^10,11^B, ^13^C, ^19^F, ^27^Al, ^29^Si, ^31^P) induces magnetic behavior, which makes them observable when using NMR. Nuclear spins are extremely sensitive to chemical and physical environments. Therefore, their NMR frequencies, which are measures of the surrounding electron densities, can provide unique information regarding the local structures of investigated materials. Furthermore, local interactions and dynamics can also be probed indirectly through the relaxation properties of induced magnetic moments. The introduction of magnetic field gradients provides an opportunity to resolve the properties described above spatially by using MR imaging (MRI). This enables researchers to follow the electrochemical and chemical processes occurring inside battery cells visually during their operation. However, the quality of collected in situ MR data can be significantly compromised by distortions in longitudinal (polarizing), and transverse (excitation), magnetic fields (B_0_ and B_1_), which are caused by the heterogeneous nature of energy storage devices [8,9].

In this paper, we review the evolution of in situ MR methods for Li-ion battery (LIB) research in terms of both cell development and pulse sequence programming. Only a combined approach will allow researchers to collect high-quality data in terms of chemical, spatial, and temporal resolution, would provide accurate quantitative information regarding various phenomena, such as mass transport in electrolytes, Li intercalation/deintercalation in active electrode materials, Li plating on anodes, and associated processes. The implementation of innovative techniques and thorough analysis of existing approaches in adjacent fields have helped researchers overcome problems that seemed to be unsolvable only a few years ago [10]. Additionally, such techniques have led to a tremendous boost in the spectral and spatial resolution of collected in situ MR data.

## 2. Electrolytes

*Pulsed-field Gradient NMR (PFG NMR) Diffusion Measurements.* The design and optimization of LIB electrolytes require methods for measuring all relevant mass-transport parameters, including ionic diffusivities and transference numbers. Low values of diffusion coefficients are often attributed to high dynamic solution viscosities and are indicative of low Li conductivity. Additionally, an Li transference number below one leads to the formation of Li salt concentration gradients in operational cells, thereby increasing their internal resistance.

PFG NMR spectroscopy [11,12] is the most commonly used tool for measuring the self-diffusion coefficients of mobile species in condensed matter. The ability to provide ion-specific information regarding transport properties is the main advantage of NMR compared to electrochemical methods. The utility of this technique is not restricted to binary electrolytes, implying that multicomponent systems can be investigated [13,14]. Additionally, ionic transport numbers ($\tau_{i}$) can be derived from PFG NMR measured diffusivity values (D_i_*) and the molar fractions of components (*x_i_*) as follows:

(1) $$\tau_{i}=\frac{x_{i}D_{i}^{*}}{\sum_{j}x_{j}D_{j}^{*}}.$$

The utilization of in situ slice-selective PFG NMR experimentation facilitates monitoring of the spatial and temporal evolution of ionic diffusivities in operating electrophoretic cells [15]. A difference of 30% in terms of Li^+^ diffusivity at opposite ends of a cell is registered by the polarization of the electrolyte under applied electric potential. However, the inability to distinguish between nuclei in neutral and charged aggregates is a well-known drawback associated with this method in an electrochemical context. Therefore, the quantity calculated by Equation (1) represents the transport number, rather than the transference number (*t_i_*), which is defined as the fraction of the total ionic current carried by all ions of a particular charge sign. The latter value must be used in the Newman model of mass transfer in electrolytes [16] and can only be replaced with *τ_i_* in cases with negligible ion pairing (i.e., very dilute solutions).

**Figure 1:**
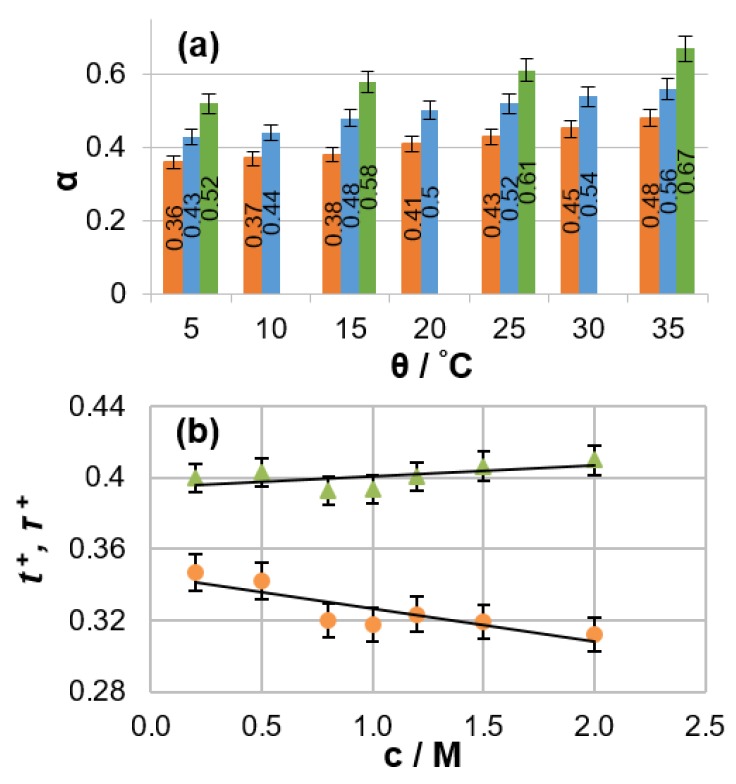
(**a**) Degree of ion pairing for 1 M LiPF_6_ in various solvent mixtures as a function of temperature. Solvent components mixed as EC:DMC (7:3 *v*/*v*, brown), EC:DMC (1:1 *v*/*v*, blue), and EC:EMC (3:7 *v*/*v*, green), where EC, DMC, and EMC are ethylene carbonate, dimethyl carbonate and ethyl methyl carbonate respectively. (**b**) Salt concentration dependence of the cation transference number *t*^+^ (brown circles) and transport number *τ*+ (green triangles) in a 1 M LiPF_6_ EC:DMC 1:1 *v*/*v* solution. (Reproduced with permission from [18]; Copyright 1948 Royal Society of Chemistry).

The PFG NMR data obtained for an electrolyte solution can be combined with the specific conductivity value measured for the same solution to calculate ensemble-averaged diffusion coefficients for positively charged, negatively charged, and neutral species [17]. The degree of ion association and the Li cation transference number can then be extracted. A detailed analysis of non-aqueous LIB electrolytes of practical interest (LiPF_6_ solutions in binary organic carbonate mixtures) was conducted using this method [18]. The dependencies of mass-transport parameters on salt concentration, temperature, and solvent composition were reported. A remarkably high degree of ion association (from 36% to 67%) was observed for the investigated electrolytes (Figure 1a). Electrolyte temperature affects ionic association in a manner similar to the salt concentration (i.e., ionic association increases with temperature or concentration). This phenomenon can be explained by the fact that the electrolyte dielectric constant, which is a measure of the ability of a substance to maintain charge separation, decreases with increasing temperature because added thermal energy disrupts the alignment of molecular dipoles in an applied electrical field. It was also shown that the discrepancy between the numerical values of lithium transference and transport numbers can be as high as 30% under typical battery operating conditions (Figure 1b).

*Electrophoretic NMR.* Although the combination of PFG NMR and conductivity measurements can provide reliable data for the parametrization of mass transfer in electrolytes, this method requires the preparation and consecutive analysis of many samples to cover the full range of conditions appearing in an operating LIB. Therefore, it would be preferable to conduct in situ experiments with operating mimics of energy storage devices to acquire a full set of data related to operation conditions in a single trial. Furthermore, NMR experiments in the presence of an electric field (electrophoretic NMR (ENMR)) can solve the problem of charged species identification and their separation from neutral ion aggregates because this technique is able to measure the velocity (*v_i_*) of ionic drift in an applied field (*E*) directly [19]. Ionic mobility (*µ_i_*) and transference numbers can be calculated from the obtained data as follows:

(2) μi=viE,

(3) $$t_{i}=\frac{x_{i}\mu_{i}}{\sum_{j}x_{j}\mu_{j}}.$$

**Figure 2:**
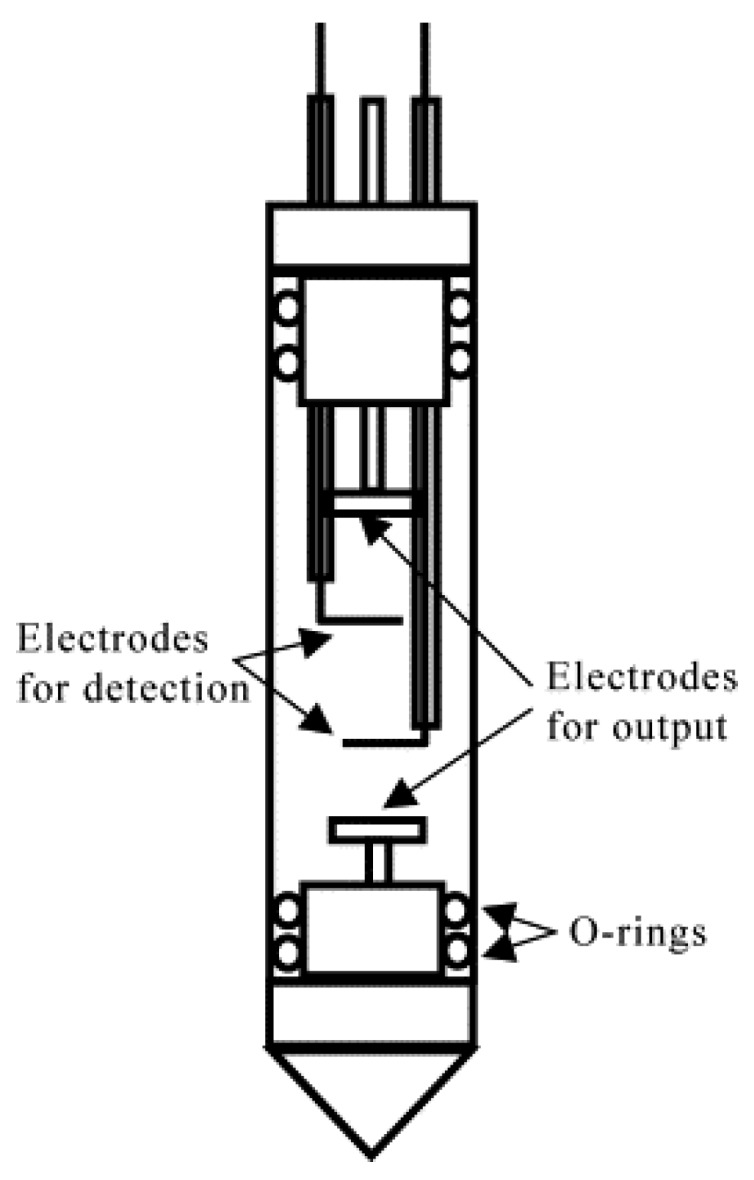
Four-terminal electrophoretic cell. The cell is composed of a cylindrical glass tube with a diameter of 10 mm. The distance between the output electrodes is 3.2 cm and that between the detection electrodes is 2.0 cm. Teflon rings are used to prevent any solution leakage. (Reproduced with permission from [21]; Copyright 2002 Elsevier).

However, there are certain complications associated with this method [20,21]. First, electric potential drop largely occurs at electrode/electrolyte interfaces because the Debye length is on the nanometer scale for electrolyte solutions at concentrations of practical interest [22,23]. Therefore, the *E* value in the active volume of NMR signal detection cannot be calculated directly based on the applied potential and electrode separation. Two additional reference electrodes must be incorporated into an electrophoretic cell to measure a voltage drop directly in the region of interest (Figure 2) [21]. Additionally, an overpotential of at least one order of magnitude greater than the electrochemical stability window of practical LIB electrolytes is required to detect the ENMR signal variations associated with ionic drift unambiguously [24,25]. This leads to continuous chemical changes in the electrolyte near the electrodes, which can affect the electric field in the active volume of the cell during experimentation. Therefore, electrodes should be placed far from the signal detection area to minimize this undesired effect. However, such cell designs with several centimeters of separation between electrodes have little in common with commercially available batteries, which decreases the practical relevance of the collected data.

*In situ MRI.* In situ MRI is a powerful alternative tool for the visualization and parametrization of mass transport in LIB electrolytes without the application of a large electrical potential for signal detection [15,26,27]. By using this technique, the spatial distribution and time evolution of the ion concentration in an electrophoretic cell under an applied electric current can be collected. Mass transport under such conditions occurs as a combination of migration and diffusion. For example, during the discharging of a cell, the electric field causes the migration of cations to the positive electrode and anions to the negative electrode. While Li^+^ ions recombine at the positive electrode with electrons that pass through the outer circuit, anions do not react on the electrode, meaning they accumulate in its vicinity. Consequently, the concentration of cations increases near the negative electrode to maintain the local electroneutrality of the electrolyte solution. This leads to the formation of an ionic concentration gradient in the electrolyte solution and a diffusion flux opposing this gradient. By combining in situ MRI experimentation with inverse mathematical modeling, one can extract spatially resolved values of the salt diffusion coefficient and cation transference number in Li-ion battery electrolyte solutions [28,29]. The spatial resolution of the obtained results is highly beneficial because the values of ionic diffusion coefficients in an electrolyte solution depend on the salt concentration. The differences in diffusivities at opposite ends of a cell can be as high as 60%, which must be considered to derive an accurate description of mass transport.

While pioneering works on the in situ MRI parametrization of electrolyte mass transport have used simple cell designs consisting of an electrolyte column held between two Li metal electrodes and a very basic spin-echo MRI pulse sequence, they have successfully demonstrated the potential of the method. However, several aspects of initial experiments had to be revised to derive more accurate and reliable data. First, the Li/Li^+^ potential is outside the electrochemical stability window of typical LIB electrolytes. This leads to continuous electrolyte decomposition with gas generation at electrode surfaces. Formed bubbles reduce the cross-sectional area of a cell, thereby increasing the current density in the corresponding regions of a sample. Furthermore, Li is deposited on the surface of the negative Li electrode in the form of mossy dendrites. While this could be useful for studying dendrite growth [8,30,31]. these experimental conditions (distance between electrodes, absence of a separator, uncontrollable pressure inside the cell, etc.) are very different from the conditions in commercially available batteries, which decreases the applicability of obtained results to actual devices. In practice, variable current density and a moving boundary of the electrolyte domain introduce undesirable complications into the inverse modeling of processes, thereby decreasing the accuracy of the obtained results.

**Figure 3:**
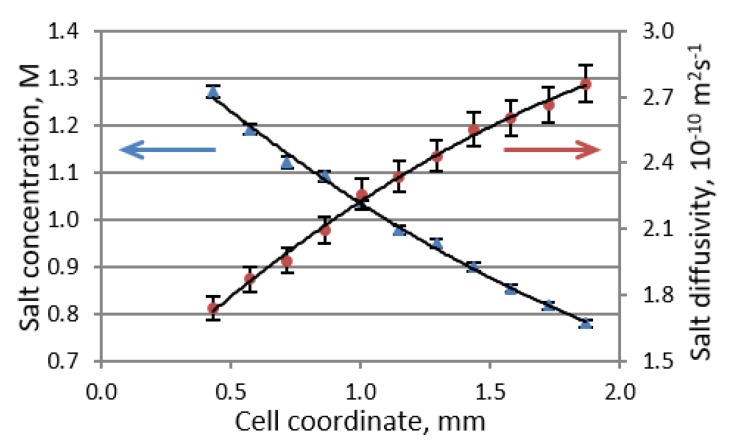
Steady-state Li salt concentration (blue triangles) and diffusivity (maroon circles) profiles in 1 M LiPF_6_/EC:DMC (1:1 *v*/*v*) at a current density of 9 A m^−2^. (Reproduced with permission from [5]; Copyright 2016 American Chemical Society).

The implementation of a graphite anode and LIB separator to an in situ cell, as well as refreshment of the electrolyte following the solid-electrolyte interphase (SEI) formation cycle, would allow one to avoid the issues associated with metallic Li. It has been demonstrated that in contrast to a symmetric cell with two Li electrodes, where continuous changes in the concentration gradient occur, a steady-state (time-invariant) condition can be achieved during the operation of Li versus a graphite cell [5]. This state occurs when the diffusion flux against the formed concentration gradient compensates for the migration flux of the ions, which can be described by the following equation:

(4) $$-D\left(c\right)\frac{\partial c}{\partial x}=\frac{\left(1-t^{+}\left(c\right)\right)J}{F},$$

where *D* is the Li salt diffusion coefficient, which is equal to the harmonic mean of the cation and anion diffusion coefficients measured by PFG-NMR, *c* is the salt concentration, *x* is the cell coordinate, *t*^+^ is the Li cation transference number, *J* is the electric current density, and *F* is Faraday’s constant. The inability to achieve a steady state in a symmetric Li versus Li cell is attributed to the ongoing formation of dendrites and continuous changes in the electrolyte domain. By combining the PFG NMR and MRI techniques into a single pseudo-3D experiment, one can determine both the salt concentration and salt diffusivity profiles of an electrolyte solution (Figure 3). The Li transference number can then be obtained by using Equation (4) according to the concentration profile that exists under steady-state conditions in the presence of an applied current.

From the pulse sequence programming perspective, basic spin-echo MRI experiments [32] were conducted in early investigations of LIB electrolytes. This is a frequency encoding technique in which MR signal acquisition occurs simultaneously with the labeling of nuclei positions in a sample based on the applied magnetic field gradient (read-out gradient). Although this is supposed to be a very time-efficient method in which an entire image can be acquired from a single scan, it is highly sensitive to magnetic field inhomogeneities, which are common in battery samples based on significant variations in the magnetic susceptibility of different cell components (e.g., the electrolyte, current collectors, and electrodes) [8,33]. As a result of these distortions, frequency encoding MRI techniques exhibit non-uniform initial electrolyte concentration profiles and “dead space” near electrodes, where the electrolyte signal is heavily suppressed. Pure phase-encoding MRI techniques provide a means of circumventing this issue for in situ cell designs [34]. Rather than allowing frequency distortions from field inhomogeneities to accrue while a signal is acquired with a read-out gradient, spatial information is encoded prior to acquisition with incremented gradients for a fixed and brief phase-encoding period. Only one image pixel can be acquired in each phase-encoding step. However, despite the additional time required to complete all gradient steps, the total acquisition time for pure phase-encoding techniques is competitive with that of frequency-encoding techniques for practical samples because an acceptable signal-to-noise can be achieved using only a small number of scans compared to frequency-encoding techniques.

**Figure 4:**
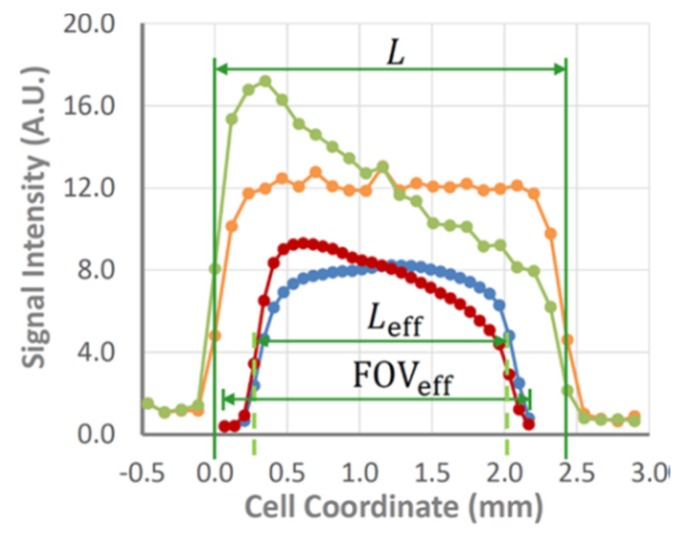
Comparison of the baseline and steady-state signal intensity profiles of 1.00 M LiPF_6_ in EC/PC/DMC, 5:2:3 (*v*/*v*) measured by chemical shift imaging (CSI) (blue and red lines; PC is propylene carbonate) and double-half k-space (DHK) single-point ramped imaging with T_1_ enhancement (SPRITE) (orange and green lines) at 10 °C with a nominal cell length of 2.43 mm, charging the graphite electrode at 7.2 A m^−2^. Horizontal compression and translation of the field of view has been applied to the CS images, illustrating the dead space captured by DHK SPRITE, but missed by CSI. (Note that the DHK SPRITE images have been vertically scaled to facilitate simultaneous plotting with the CS images). (Reproduced with permission from [39]; Copyright 2017 American Chemical Society).

Chemical shift imaging (CSI) [35] is one of the simplest pure phase-encoding techniques. It avoids the blurring associated with spins resonating at different frequencies by localizing different pixels and can be readily coupled with a shift- or slice-selective excitation pulse, as well as diffusion or relaxation measurements [5,36]. While CSI tolerates B_0_ inhomogeneity caused by magnetic susceptibility variations, it suffers from distortions in the radio-frequency B_1_ field near conductive parts of a battery, resulting in phase and amplitude alterations of the excitation and inversion pulses. Residual eddy currents, which are generated in the metallic components of a cell and probe following the application of large phase-encoding gradient pulses, are another known issue in CSI that can lead to potential signal corruption. As a result, a dead space at the edges of the electrolyte domain near the electrodes is still observed in CSI images. However, it is an order of magnitude narrower than that observed in the case of frequency-encoding imaging. The double-half k-space (DHK) single-point ramped imaging with T_1_ enhancement (SPRITE) [37] technique combines ramped gradients with single-point, low-flip-angle imaging. The former helps to suppress residual eddy currents substantially while the latter makes a sequence insensitive to B_1_ inhomogeneity near metallic surfaces (Figure 4) [38,39]. However, there is a tradeoff because DHK SPRITE is a low-flip-angle technique, meaning its signal-to-noise ratio is lower than that of CSI. Therefore, many scans are required to achieve clear data, meaning this method is mostly suitable for acquiring baseline and steady-state images. In contrast, relatively smooth CSI images can be acquired rapidly enough to capture the transient behaviors of the concentration gradient building up to the steady state. The robustness of DHK SPRITE against inhomogeneity variations and field distortions near the electrodes provides a means of accurately measuring a cell and capturing the dead space near electrodes, which negatively impacts CSI. A combination of CSI and DHK SPRITE was utilized for a detailed study and performance comparison of two electrolytes (1M LiPF_6_ dissolved in either a mixture of EC/DEC 1:1 *v*/*v* or EC/PC/DMC 5:2:3 *v*/*v*) in a temperature range of 10–40 °C. Temperature was shown to have a significant influence on the steady-state concentration gradient, as well as the rate of its buildup. Additionally, it was determined that a conventional 1.00 M LiPF_6_-EC/DEC (1:1 *v*/*v*) mixture generated salt precipitation under polarization at 10 °C. The loss of salt under strong polarization at low temperatures is a potential source of long-term capacity fading in Li-ion batteries.

*Solid electrolytes (SE).* The replacement of commonly used organic-solvent-based liquid electrolytes by solid-state alternatives would allow to avoid many safety concerns associated with their flammability, toxicity, and high reactivity. SE also can serve as a physical barrier to dendritic growth, enabling utilization of Li metal instead of conventional graphite anodes. A shift to Li metal with an extremely high theoretical specific capacity, low mass and the lowest negative electrochemical potential would offer an increase of up to 70% in energy density of LIBs.

Many publications regarding NMR analysis of structural features and Li dynamics characterization in various solid ion conductors have been reported recently. However, most of those data were collected ex situ [40,41,42]. which could be partially explained by the novelty of the task. Fundamental questions regarding the structural composition and functionality of newly developed materials should be answered before an investigation of degradation mechanisms during application of those materials in LIB with in situ techniques. “Classical” well-established experiments can provide the required information. For example, a comprehensive solid-state NMR analysis was carried out to reveal the local structure and mobility of lithium ions in a series of Li_1+x_Al_x_Ti_2−x_(PO_4_)_3_ (LATP) materials with 0 ≤ x ≤ 1 [43]. Monitoring of the structural symmetry loss during the process of the Al-substitution with the complete site assignments was done using the multinuclear (^6,7^Li, ^27^Al, and ^31^P) NMR. Spin-lattice relaxation rates analysis as well as ^7^Li NMR spectral lines narrowing indicated diffusion-induced maxima when 0.35 ≤ x ≤ 0.5. Slowly moving Li ions in additional phosphate phases formed at the expense of LATP when x is higher than 0.5 can be proposed based on ^6^Li exchange spectroscopy data.

Lithium diffusivity in solid electrolytes is typically 2–3 orders of magnitude slower than in liquids, however, implementation of diffusion probes providing strong gradient pulses makes PFG NMR experiments in solids possible, when the NMR signal relaxation is not extremely fast. For example, garnet-type Li_6.5_La_3_Zr_1.5_Ta_0.5_O_12_ (*D*_Li_ = 1.9 × 10^−13^ m^2^ s^−1^ at 298 K) [44], anti-perovskite type Li_2_OHCl (*D*_Li_ = 6.0 × 10^−13^ m^2^ s^−1^ at 373 K) [45], and halide-rich argyrodites Li_5.5_PS_4.5_Cl_1.5_ (*D*_Li_ = 1.0 × 10^−11^ m^2^ s^−1^ at 300 K) [46] were studied that way.

NMR sensitivity to both ^6^Li and ^7^Li isotopes opens a unique opportunity to investigate Li ion pathways within solid electrolytes using isotope exchange. A composite electrolyte consisted of Li_7_La_3_Zr_2_O_12_ (LLZO) garnet and polyethylene oxide (PEO) was placed in between two ^6^Li-labeled lithium foils and ion transport was monitored by the ^6^Li → ^7^Li isotope replacement during the cycling of the cell as ^6^Li ions partially substitute ^7^Li ions inside the electrolyte every time they move through it [47,48]. It was demonstrated that Li ions favor the pathway through the LLZO ceramic phase instead of the PEO-LLZO interface or PEO. The experiments were carried out ex situ, meaning that ^6^Li and ^7^Li NMR spectra were compared for pristine electrolyte and for the electrolyte extracted from the cycled cell. It was done in order to avoid sample inhomogeneity caused by the presence of various battery components, and to obtain the best spectral resolution possible for that material. In principle, this approach can be extended to in situ application, with discrimination of signals by their relaxation times in case of significant overlapping [38].

A possibility of in situ NMR characterization of all solid-state batteries was demonstrated recently for a Li–Li_6.5_La_3_Zr_1.5_Ta_0.5_O_12_ (LLZTO)–Li cell [49]. ^7^Li CSI images were collected at different stages of the cell cycling. Surface transformations at both the stripping and plating interfaces, indicating heterogeneities in both Li removal and deposition were observed in these experiments. Moreover, despite the original assumption that SE would provide a physical barrier to dendritic growth, dense Li microstructures that penetrate into the electrolyte pellets and ultimately lead to the cell death are seen in the images before any short-circuits are observed in the voltage profiles during electrochemical measurements.

## 3. Anodes

*Graphite.* Graphite electrodes, which have been used in LIBs since their commercialization, are still the most relevant negative electrodes for EV batteries [50]. Stoichiometric and thermodynamic stability combined with good in-plane electronic conductivity are important features of this material that make it attractive for LIB use. Li intercalation into graphite is accompanied by stage-like changes in the average spacing of graphene planes, yielding a rich phase diagram containing distinct plateaus in a voltage versus Li concentration plot [51]. Phases can be assigned to the dilute (1′, 4, 3, and 2L) and concentrated (2 and 1) stages. The first four phases correspond to Li_1/12_C_6_, Li_1/6_C_6_, Li_2/9_C_6_, and Li_1/3_C_6_ stoichiometry and demonstrate liquid-like disorder, whereas the latter two phases (Li_1/2_C_6_, LiC_6_) exhibit solid-like order.

The electrochemical lithiation/delithiation of graphite was followed by real-time in situ NMR using a lithium/graphite electrochemical cell [52,53]. A free-standing graphite electrode and Li-metal foil were pressed into copper grids, laminated together using an electrolyte-soaked separator, and then sealed in a plastic bag. The cell was cycled in galvanostatic mode at a current rate of C/20 inside an NMR magnet. Spectroscopic signatures, such as chemical shifts and quadrupolar satellites, were identified for each phase of graphite lithiation. The dilute phases possess the central line and are distinct from the concentrated phases (0–18 ppm and 42–45 ppm, respectively). Signal assignment within the stages types is more nuanced and requires detailed comparisons of the central line chemical shift and quadrupolar splitting. Overall, it was demonstrated that NMR is very sensitive to the structural changes within graphite associated with its lithiation and can serve as an external reference for determining an electrode’s state of charge (SOC). These data are also in good agreement with previously reported ex situ NMR spectra [54].

The central line width of a ^7^Li NMR signal also depends on the degree of graphite lithiation, and shows its maximum in stage 2. The line width is inversely proportional to the NMR signal transverse relaxation time (T_2_), which depends on the spin mobility within the studied material. Therefore, it was concluded that Li diffusion in graphite varies with the SOC ($D_{{\mathrm{Li}}_{1/2}C_{6}}<D_{{\mathrm{LiC}}_{6}}<D_{{\mathrm{Li}}_{1/3}C_{6}}$). This observation is in good agreement with data obtained using a potentiostatic intermittent titration technique, where it was determined that the chemical diffusion coefficient of Li in graphite depends heavily on the amount of intercalated Li [55,56]. In particular, a local minimum with an order of magnitude decrease in Li diffusivity can be observed at the beginning of the stage-2L-to-stage-2 transformation. One would expect that such a phenomenon would have an effect on the Li distribution within graphite during battery cycling. In situ MRI can visualize and quantify this process. However, until recently, MRI investigations of Li intercalation into the active material particles of an electrode were significantly hampered by the extremely fast decay of Li signals (T_2_ ≤ 100 μs). In classical imaging techniques, a period of 500 to 2000 µs is required to turn the spatial encoding gradient pulse on, let the field stabilize, label the transverse magnetization of the observed nucleus, turn the pulse off, and let the field stabilize again. This period is too long and the signal disappears before it can be observed.

**Figure 5:**
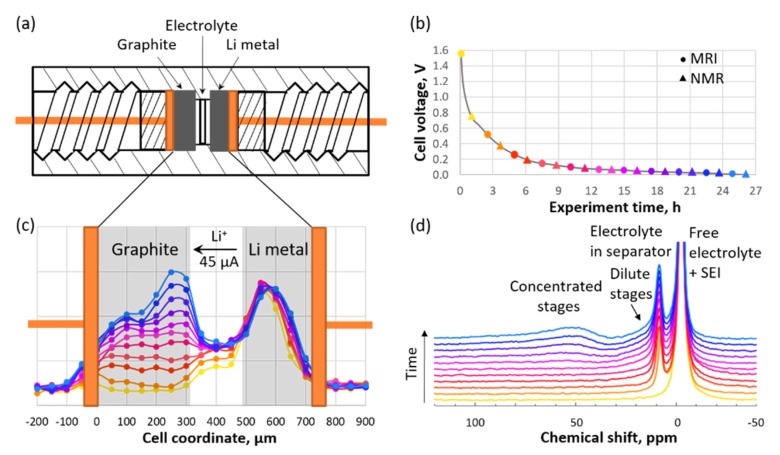
(**a**) Schematic representation of an in situ cell. (**b**) Voltage vs. time curve for graphite during the first charging stage. (**c**) Axial ^7^Li MR images. (**d**) ^7^Li NMR spectra collected during charging. The circles of a given color in panel (**b**) correspond to the curves with the same colors in panel (**c**), while the triangles correspond to the curves with the same colors in panel (**d**). (Reproduced with permission from [57]; Copyright 2018 American Chemical Society).

DHK SPRITE is capable of acquiring images of fast-relaxing materials because both radio frequency (RF) excitation and data acquisition occur while the encoding gradient is turned on, meaning field stabilization delays are not required and the time between excitation and acquisition can be on the order of tens of microseconds. This method also yields images free of distortions caused by magnetic field inhomogeneities, susceptibility variations, and nuclear spin interactions, therefore it seems to be a natural choice for the in situ mapping of Li distributions within an electrophoretic cell. However, considering that the acquisition in DHK SPRITE occurs when the magnetic field gradient is turned on, it is impossible to collect spectroscopic information regarding the species under investigation. Imaging must be accompanied by standard NMR spectroscopy to overcome this limitation (Figure 5).

Experimentally determined Li concentration profiles within a 300-μm-thick graphite electrode during cell operation were reported recently [57]. The proposed method provides a spatial resolution of 50 μm. It was demonstrated that initial graphite lithiation occurs relatively uniformly, leading to a flat concentration profile. This phenomenon can be explained by the decreasing equilibrium overpotential curve at an early SOC. If a certain particle becomes more lithiated than the surrounding particles, then its overpotential decreases, meaning insertion into that particle is no longer favorable. However, once the value of the SOC reaches approximately 0.22, which occurs roughly simultaneously throughout the electrode, the graphite reaches a 100 mV plateau and transforms from Li_2/9_C_6_ into Li_1/2_C_6_ through Li_1/3_C_6_. The incentive for Li to fill in uniformly across the breadth of the electrode is removed by the flattening of the overpotential. This flattening coincides with a significant decrease in the solid-state diffusivity at SOC = 1/3. The formation of a steep concentration gradient on the edge of the graphite electrode closest to the separator can be detected using MRI. A phase transformation from stage 2L to stage 2 begins in this area and proceeds until its completion, followed by the appearance of the next energy barrier and a diffusion drop off. Next, the reaction front moves toward the deeper regions of the electrode near the separator, as indicated by the concentration profile. At the point when the cell reaches the 2 mV cutoff potential, the layer of the graphite electrode closest to the separator is highly lithiated at SOC = 0.77, whereas the slice closest to the current collector is only at SOC = 0.30. Unfavorable Li dynamics during the phase transformations of graphite lead to side reactions at particle interfaces. The effects of these side reactions can be minimized by a proper charging protocol [57].

*Li-titanate (Li_4_Ti_5_O_12_, (LTO)).* Based on a lack of volume changes during lithiation, which is essential for an extremely long operational battery lifetime, coupled with improved safety resulting from extremely flat discharge and charge plateaus, Li-titanate is considered to be an extremely useful electrode material [58]. The presence of paramagnetic Ti^(III)^ during the charging of LTO to Li_7_Ti_5_O_12_ makes it difficult to analyze this material using in situ NMR because the interactions of ^7^Li nuclei with unpaired electrons significantly reduces the relaxation times of NMR signals, making the peaks in the spectrum exceedingly broad and difficult to observe. Spinning a sample under magic angle conditions (MAS NMR) can suppress the anisotropic (orientation-dependent) part of this interaction [59], making the signals narrower and easier to analyze. However, until recently, only static cells were used for in situ battery NMR experiments (in situ cells designed for MAS NMR will be described later in the cathode section of this review), largely limiting the NMR characterization of LTO to ex situ techniques [60,61]. It was determined that while a pristine material contains Li at 8a and 16d sites (^7^Li NMR signals are located at 0.08 ppm and −0.26 ppm, respectively), a broad resonance corresponding to Li located at 16c sites (−10 ppm) appears during LTO lithiation. In addition to direct Li insertion into 16c sites, the migration of Li between 8a and 16c sites was also observed. These two processes lead to the eventual phase transformation of spinel Li_4_Ti_5_O_12_ into rock salt Li_7_Ti_5_O_12_ at the conclusion of LTO lithiation. The mechanism is reversible during delithiation [61].

An elegant method for collecting spatially resolved information regarding Li distributions in an operating Li_x_CoO_2_ (LCO)/LTO electrochemical cell was reported by Tang et al. [62]. The suggested scanning image-selected in situ spectroscopy technique takes advantage of the considerably longer longitudinal relaxation time (T_1_ > 5 ms) compared to the transverse relaxation time (T_2_ < 0.1 ms) of ^7^Li NMR signals corresponding to intercalated Li. A selective inversion experiment with an adiabatic 180° pulse performed in the presence of a pulsed field gradient allows one to acquire ^7^Li NMR spectra within 100-µm-thick slices. Magnetization is stored along the z axis during the gradient pulse, followed by a stabilization delay of 500 µs. It is then transferred to the x-y plane by a 90° pulse for signal acquisition. While the spectral resolution of static spectra is insufficient for separating peaks corresponding to various Li sites, the evolution of the median position and width of the total signal closely match the Li_4_Ti_5_O_12_ to Li_7_Ti_5_O_12_ transformation. An in situ visualization of the lithiation front in thick electrodes (approximately 500 µm) during cell charging/discharging was achieved with a spatial resolution of 100 µm.

*Silicon.* Although graphite is currently the primary anode material used in commercial LIBs, extensive research efforts have been devoted to the investigation of silicon materials as potential replacements. A superior gravimetric capacity of over 3000 mAh/g is a very attractive benefit of silicon compared to graphite (372 mAh/g) [50]. However, dramatic volume expansion (approximately 300%) during lithiation leads to the separation of silicon particles from the electrically conductive carbon matrix after multiple cycles, which is a significant drawback [63]. Additionally, this physical instability disrupts the solid electrolyte interphase that forms on the surface of silicon particles, promoting continued breakdown and loss of the electrolyte. Many studies have attempted to gain a detailed fundamental understanding of the electrochemical lithiation of silicon [6,64,65]. The availability of NMR for characterizing both crystalline and amorphous materials makes it an ideal technique for the investigation of silicon-based anodes because the electrochemical cycling of silicon involves a number of amorphous phases.

In situ ^7^Li NMR analysis of a Li/Si battery with a baggy cell design identical to that used for graphite studies revealed that Li initially reacts with carbon in the Si/C composite electrode in the early stages of electrode lithiation [6]. Immediately following this reaction process, additional peaks corresponding to the formation of small silicon clusters and isolated Si atoms were detected in the spectrum. This continuing process is accompanied by further breakage of the Si network and the appearance of isolated Si atoms. Another resonance appears in the spectrum near the end of lithiation. This signal was attributed to an overlithiated crystalline phase (*c*-Li_15+δ_Si_4_). This excess Li phase is extremely reactive in the electrolyte and the Li/Si cell “self-discharges,” leading to the loss of Li from this phase and an accompanying increase in open circuit voltage. The strong reactivity of *c*-Li_15+δ_Si_4_ prevents its detection using ex situ NMR analysis because it is completely reacted during the time between battery disassembly and NMR experimentation, which highlights the importance of performing in situ measurements.

**Figure 6:**
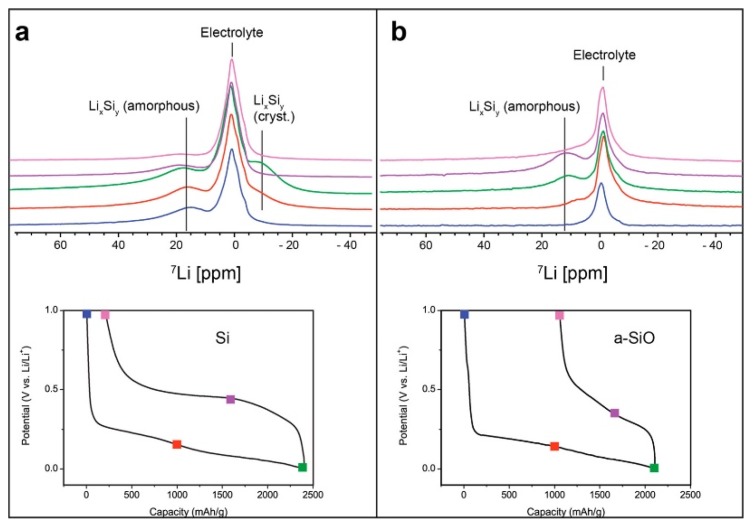
Comparison of ^7^Li in situ snapshots detailing changes in the Li chemical shift for cellulose-based silicon metal anodes (**a**) and a-SiO (**b**). (Reproduced with permission from [66]; Copyright 2019 American Chemical Society.

A novel jelly-roll-type in situ cell design was recently developed and utilized for a comparative study of silicon and amorphous silicon monoxide (a-SiO) anodes [66]. The copper current collector of the anode in the cell was replaced with a porous cellulosic substrate, which introduces noteworthy advantages for the NMR spectral analysis of electrode materials. First, the improved adhesion of electrode slurries using water-based binders onto porous substrates allows one to increase the active material loading of the electrode. Second, a porous structure facilitates the utilization of multiple electrode layers in a cell, where the electrolyte can flow through the pores in cellulosic substrates. Although this reduces the effective cycling rate attainable by increasing ionic resistance, it dramatically increases the amount of active material. For low-sensitivity nuclei, including ^29^Si, the resulting signal boost can be particularly advantageous. Finally, the lack of a metallic current collector improves electromagnetic field homogeneity and RF permeability in the cell. The lithiation of a-SiO exhibited some significant differences compared to the lithiation of pure Si. For example, a crystalline Li_15+δ_Si_4_ phase was not detected at full charge (Figure 6). The SiO_2_ domains in a-SiO initially break down to form irreversible Li silicates and reversible Li silicides, as indicated by ^29^Si MAS NMR analysis. The Knight shift of the silicides formed from a-SiO are much less apparent than those in bulk Li silicides, supporting the hypothesis of the formation of fewer metallic nanoscale amorphous domains of Li_x_Si_y_ during lithiation. Upon delithiation, the silicides revert back to an amorphous silicon structure.

The utilization of silicon nanocomposites, such as Si nanowire (SiNW), can help accommodate the volume expansion of an electrode during lithiation and the subsequent loss of electrical connections. A SiNW-based model anode system for the in situ NMR baggy cell was prepared by growing SiNWs on a commercial carbon support [67]. The assembled cell demonstrated significantly enhanced cyclability, facilitating the monitoring of amorphous and amorphous-to-crystalline Li-silicide transformations in the Li/Si cell beyond the first cycle.

*Li-plating.* A significantly longer “refueling” time for EVs compared to standard internal combustion engine vehicles is a substantial disadvantage from an end user’s perspective. An increase in charging current by a factor of three is required to reduce this gap and promote the widespread adoption of EVs on the mass market [68]. Li plating on the negative electrode is a drawback for fast charging. If the current exceeds the intercalation rate at which Li diffuses into the negative electrode, then a film of metallic Li will deposit on the electrode surface. The plated metal then reacts with the electrolyte, leading to an increase in cell internal resistance, capacity fading, and potential short circuiting in extreme cases [69]. Li can form either large dense particles or mossy microstructures and can re-intercalate into the graphite anode at a later time. This high reactivity has necessitated the development of in situ methods for accurately characterizing the Li plating process.

As mentioned previously, NMR provides distinct spectroscopic signatures for various stages of graphite lithiation and metallic Li. Additionally, NMR is also sensitive to the orientation of the metal surface toward the static magnetic field, exhibiting a higher shift for surfaces parallel to B_0_ than for those orthogonal to the field [49,70,71]. Special attention should be paid to proper in situ cell design because multiple factors, including quality of materials, cell geometry, temperature, and the cell’s SOC, can influence the Li plating phenomenon. Minimization of the metallic components of the cell, such as the substitution of foil current collectors with mesh or wire collectors, is a typical approach to in situ NMR cell design for reducing field distortions [66,72,73]. However, such alterations of standard battery materials can generate a non-uniform electric current distribution through electrodes and can stimulate additional Li plating, thereby decreasing the relevance of the obtained results for battery manufacturers.

The in situ NMR characterization of Li plating in single-layer pouch cells comprising actual components of positive electrodes (LiCoO_2_, LiNi_x_Co_y_Al_z_, and LiMn_2_O_4_) and negative electrodes (graphite and hard carbon) sealed by Al-deposited laminate films was reported recently [74]. A preliminary study revealed that even though the NMR signal of battery components was attenuated by up to 35% to 40% of the original intensity based on RF shielding by the laminate and metallic current collectors, it was still sufficiently strong for practical application. It was assumed that the signal was obtained only from the “edge” parts of the cells and traveled out through voids between deposited aluminum particles in the laminate films. The cells were charged up to 170% SOC using 2C or 3C electric current and the evolution of the Li metal resonance was monitored using ^7^Li in situ NMR. The measurements revealed a “relaxation effect,” meaning a decrease in the signal attributed to Li metal deposited on the negative electrode surface, based on overcharging. The reduction in the Li metal signal was inversely proportional to the increase in the signal of Li stored in carbon. Therefore, this effect was attributed to absorption of deposited Li into the carbon of negative electrodes. Furthermore, the influence of temperature and battery operating conditions on Li metal deposition was investigated using the same cell setup [75]. Similar activation energies were obtained for the Li metal deposition rate and cell capacity fading rate, suggesting that Li metal deposition is the main cause of capacity fading during low-temperature cycling.

The accuracy of obtained results heavily depends on the homogeneity of electromagnetic fields (B_0_ and B_1_), which can be significantly compromised by the presence of conductive elements in a battery. In particular, the attenuation of an RF field on metallic Li will lead to a difference in the magnetization flip angles for Li metal and intercalated Li resonances, which complicates the direct comparison of their intensities in collected spectra. NMR probe design, which can facilitate favorable field orientation to avoid the distortion of B_0_ and cancelation of B_1_ in current collectors, can help minimize field inhomogeneity issues. Ideally, both fields should be orthogonal to each other and parallel to the planes of the electrodes. The concept of a resonator consisting of two parallel plates and a flat sample inserted in between the plates was developed for the MRI of histological tissues [76]. The efficiency of such a resonator was demonstrated by monitoring water content and water transport in proton electrolyte membrane fuel cells [3,77]. Assembling such a probe directly on the outside of a single-layer prismatic cell would allow researchers to obtain the desired field alignment. More recently the potential of a similar approach for magnetic resonance experiments on lithium ion cells have been demonstrated utilizing a model system [78].

## 4. Cathodes

In situ NMR studies of cathodes are particularly sparse because cathode materials typically produce much broader line shapes based on their paramagnetic nature, meaning they are more difficult to probe. A broad resonance from −100 to 100 ppm was reported for a fresh LCO positive electrode [79]. The oxidation of diamagnetic Co^3+^ into paramagnetic Co^4+^ occurred during the electrochemical delithiation of the cathode to maintain the charge balance. This led to the appearance of an even broader ^7^Li NMR signal in the spectrum at approximately 90 ppm with a simultaneous decrease in the original peak at 0 ppm. This large reduction in the total signal intensity at the beginning of charging (approximately half of its original intensity during the extraction of only 3% of the Li) was attributed to the localized nature of the phase transformation at this SOC. The intensity recovered back to its expected value when the distribution of Co^4+^ became more uniform. It was demonstrated that for x ≤ 0.75, the signal reduction was consistent with the Li content in LCO estimated from the electrochemical measurements. Furthermore, it was determined that the ^7^Li NMR chemical shift in Li_x_CoO_2_ correlated well with the distance between CoO_2_ layers, where Li^+^ ions were located. This demonstrated the power of in situ NMR for the quantitative analysis of Li inventory in an operating battery. In particular, it was shown that LCO was not fully lithiated by the end of the cycle. A Li_0.97_CoO_2_ stoichiometry was observed instead. This small amount of lithium deficiency in LCO cannot be detected using X-ray diffraction and neutron diffraction analysis.

A much broader signal covering the region from −500 to 1500 ppm was reported for the in situ ^7^Li NMR of a Li_1.08_Mn_1.92_O_4_ (LMO) cathode [80]. This resolution is insufficient for extracting any meaningful spectroscopic information from the collected data. Relaxometry analysis was conducted, which facilitated the characterization of Li motion during the electrochemical lithiation/delithiation of the material. The T_2_^*^ values measured during the in situ experiments significantly depended on the Li content in the cathode, exhibiting smooth and nonlinear variation with a local maximum at approximately 50% SOC. This was attributed to reduced Li motion associated with a solid-solution phase transformation at this state, which correlated well with the appearance of a local minimum in the Li diffusion coefficient calculated using Monte Carlo simulations based on the lattice gas model. It was noted that the T_2_^*^ behavior differed between charging and discharging, indicating differences in the degree of ordering and structural pathways during the delithiation and lithiation of LMO.

The optimal measuring conditions for an NMR resonator can change during in situ experiments according to variations in battery material properties during cell cycling. For example, new microstructures may form or a non-metallic component may become metallic. A new automatic tuning matching cycler in situ NMR probe system was developed recently to maintain ideal resonance conditions throughout an experiment and eliminate signal attenuation caused by the loss of probe sensitivity [81]. A plastic bag cell with a LiFePO_4_ (LFP) cathode and metallic Li anode was selected as a test sample. Although the ^7^Li NMR signal of LFP is extremely broad (700 kHz, corresponding to 6000 ppm in a 7T spectrometer) and completely overlapped with the Li metal and electrolyte peaks, the ^31^P resonances of LiFePO_4_ and LiPF_6_ were well separated. This method provides an additional source of NMR information and allows researchers to track the phase transformation of LFP during electrochemical cycling.

**Figure 7:**
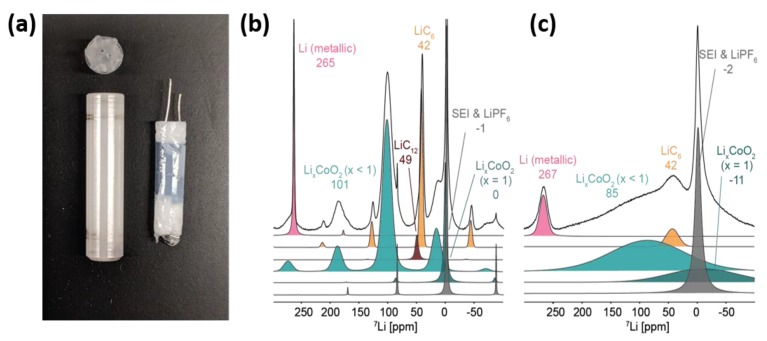
(**a**) Commercial 4 mm NMR rotor (left) and assembled jelly-roll battery insert (right). Deconvolution of the ^7^Li NMR spectra of a fully charged LiCoO_2_/graphite cell under (**b**) magic angle spinning (MAS) speed of 10 kHz and (**c**) static conditions. (Reproduced with permission from [82]; Copyright 2019 American Chemical Society.

As mentioned in the LTO section of this review, the broadening associated with paramagnetic sites can be reduced through MAS. However, designing a cell suitable for MAS comes with significant challenges. For example, when performing MAS inside a strong magnetic field, the conductive metals in a battery can produce eddy currents, causing spinning stability issues. The jelly-roll-type in situ cell design with a porous cellulosic substrate serves as a matrix for active electrode materials, which was mentioned above for the a-SiO study [66], can be modified to fit inside a 4 mm MAS NMR rotor (Figure 7a). Such a cell geometry has rotational symmetry superior to that of other battery designs, making it more suitable for a spinning cell. As a proof of concept, the charge and discharge cycle of a jelly-roll Li-ion cell containing a LiCoO_2_ cathode and graphite anode was analyzed using ^7^Li MAS NMR at a spinning speed of 10 kHz [82]. A tremendous boost in spectral resolution was achieved by introducing MAS (Figure 7b,c). This method allows researchers to monitor both anode and cathode electrodes concurrently, which is valuable for tracking the Li distribution in a full cell in real time and can also facilitate the identification of causes of capacity loss (Li plating and SEI growth) that are not readily available from bulk electrochemical analyses and other post-mortem strategies.

## 5. Future Directions

Further development of in situ NMR for battery application should focus on adaptation of the technique to the characterization of real industrial batteries instead of dealing with significantly simplified cells ignoring multiple factors influencing the cell performance. An inside-out imaging approach was reported recently, when it was demonstrated that the monitoring of magnetic field perturbation around the battery could provide reliable information about the design, state of charge, accumulated mechanical defects, and manufacturing flaws of the device [83,84]. Of course, in reality, a combination of factors could be involved into battery degradation, and each of them would affect the field distribution. Therefore, it could be difficult to separate and to identify them from the complex picture, but a possibility to use NMR for monitoring a battery designed for the commercial device is intriguing. On top of that, few would disagree that utilization of permanent (induced) magnetic field produced by the cell is a very elegant way to solve the problem of RF field penetration through the polymer-lined aluminum cell casing.

The utilization of low-frequency nuclear magnetic resonance produced by single-sided NMR sensors constructed on permanent magnets could be an alternative approach to overcome the issue caused by the aluminum foil presence in the cell cover [85]. This would provide a direct visualization of processes inside the cell in contrast to the indirect technique described above. While this approach has not been applied to batteries yet, it was used to noninvasively and nondestructively validate quality of tomato concentrate stored in >200 L metal-lined containers [86]. Of course, spectral resolution of collected that way spectra will be very low, but using relaxometry (T1, T2) and diffusion measurements techniques one should be able to detect such battery degradation processes as electrolyte decomposition and drying out, Li dendrites growth, or dissolution of transition metals from electrodes to electrolyte.

## 6. Conclusions

In situ NMR and MRI are powerful techniques for collecting ion-specific information regarding material evolution and charge distribution inside an operating Li-ion battery. Recent development of in situ MAS cells and high-resolution electrode imaging, which existed only in form of concepts just few years ago, has significantly enhanced capabilities of the method and promoted its application to the investigation of energy storage devices during electrochemical cycling. These advancements have been achieved through the thorough analysis of existing methods in adjacent areas of material research and the implementation of innovative approaches to experimental design. Further development should focus on the applicability of such tools to the characterization of real industrial batteries and to the detection and investigation of their failure mechanisms.
